# Dicaesium diaqua­bis­(methyl­ene­diphospho­nato-κ^2^ *O*,*O*′)cobaltate(II)

**DOI:** 10.1107/S1600536811035355

**Published:** 2011-09-14

**Authors:** Kina van Merwe, Hendrik G. Visser, Johan A. Venter

**Affiliations:** a Department of Chemistry, University of the Free State, PO Box 339, Bloemfontein 9330, South Africa

## Abstract

The asymmetric unit of the title compound, Cs_2_[Co(CH_4_O_6_P_2_)_2_(H_2_O)_2_], is comprised of one bidentate methyl­enediphospho­nate ligand and one water mol­ecule which are coordinated to the Co^II^ atom, as well as a caesium counter-cation. The Co atom occupies a special position on a crystallographic inversion center. The caesium ion is octa­hedrally coordinated by six O atoms with Cs—O distances ranging from 3.119 (2) to 3.296 (2) Å. A three-dimensional network is formed through O—H⋯O hydrogen bonds.

## Related literature

For related structures, see: Fleisch (1991 ▶); Neville-Webbe *et al.* (2002 ▶); Van der Merwe *et al.* (2010 ▶). For bond lengths and bond angles in related structures, see: Bao *et al.* (2003 ▶); Cao *et al.* (2007 ▶); Gong *et al.* (2006 ▶); Van der Merwe *et al.* (2009 ▶); Visser *et al.* (2010 ▶); Yin *et al.* (2003 ▶).
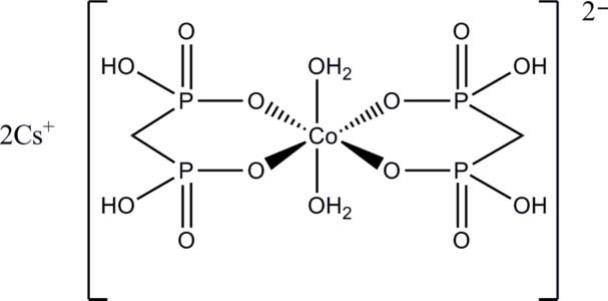


## Experimental

### 

#### Crystal data

Cs_2_[Co(CH_4_O_6_P_2_)_2_(H_2_O)_2_]
*M* *_r_* = 708.75
Triclinic, 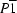

*a* = 7.333 (5) Å
*b* = 7.412 (5) Å
*c* = 7.666 (5) Å
α = 74.621 (5)°
β = 83.064 (5)°
γ = 86.496 (5)°
*V* = 398.6 (5) Å^3^
*Z* = 1
Mo *K*α radiation
μ = 5.96 mm^−1^
*T* = 293 K
0.38 × 0.07 × 0.05 mm

#### Data collection

Bruker APEXII CCD diffractometer
Absorption correction: multi-scan (*SADABS*; Bruker, 2001 ▶) *T*_min_ = 0.210, *T*_max_ = 0.755
4203 measured reflections
1904 independent reflections
1827 reflections with *I* > 2σ(*I*)
*R*_int_ = 0.015

#### Refinement

*R*[*F* ^2^ > 2σ(*F* ^2^)] = 0.015
*wR*(*F* ^2^) = 0.044
*S* = 0.75
1904 reflections
132 parameters
11 restraints
H atoms treated by a mixture of independent and constrained refinement
Δρ_max_ = 0.47 e Å^−3^
Δρ_min_ = −0.48 e Å^−3^

### 

Data collection: *APEX2* (Bruker, 2005 ▶); cell refinement: *SAINT-Plus* (Bruker, 2004 ▶); data reduction: *SAINT-Plus*; program(s) used to solve structure: *SHELXS97* (Sheldrick, 2008 ▶); program(s) used to refine structure: *SHELXL97* (Sheldrick, 2008 ▶); molecular graphics: *DIAMOND* (Brandenburg & Putz, 2005 ▶); software used to prepare material for publication: *WinGX* (Farrugia, 1999 ▶).

## Supplementary Material

Crystal structure: contains datablock(s) global, I. DOI: 10.1107/S1600536811035355/jh2322sup1.cif

Structure factors: contains datablock(s) I. DOI: 10.1107/S1600536811035355/jh2322Isup2.hkl

Additional supplementary materials:  crystallographic information; 3D view; checkCIF report

## Figures and Tables

**Table 1:** Hydrogen-bond geometry (Å, °)

*D*—H⋯*A*	*D*—H	H⋯*A*	*D*⋯*A*	*D*—H⋯*A*
O1—H1*A*⋯O4^i^	0.92 (2)	1.94 (2)	2.860 (3)	173 (3)
O5—H5*B*⋯O4^ii^	0.85 (2)	1.69 (3)	2.518 (3)	164 (7)

Symmetry codes: (i) 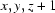; (ii) 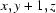.

**Fig. 1.**
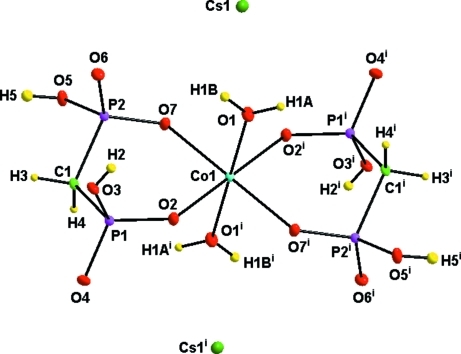
Representation of the title compound, showing the numbering scheme and displacement ellipsoids drawn at the 50% probability level. [Symmetry code: (i) 1 - x, -y, 1 - z].

## supplementary crystallographic information

### Comment

This work is part of an ongoing investigation aimed at synthesizing and characterizing new methylene diphosphonate complexes and expanding on our knowledge of the interactions of the methylene diphosphonate ligand with various metal centers. (Van der Merwe *et al.* (2009) & Van der Merwe *et al.* (2010)).

Methylene diphosphonates (O_3_PCH_2_PO_3_) has a diversified coordination capability with metal ions, due to the single methyl group which divides the two phosphonate groups. The formation of a stable six-membered ring comprised of M—O—P—C—P—O is favoured (Bao *et al.* (2003). Bisphosphonates adhere strongly to hydroxyapatite crystals and constrain their formation and dissolution (Fleisch (1991)). This physicochemical *in vivo* effect may result in the prevention of soft tissue calcification or even prevent normal calcification. The bis(phosphonic acid) has a high affinity for bone surfaces and it is also non-hydrolyzable (Neville-Webbe *et al.* (2002).

The asymmetric unit of the title compound, Cs_2_[Co(CH_4_O_6_P_2_)_2_(H_2_O)_2_], is comprised of one bidentate methylene diphosphonate ligand and one water molecule which are coordinated to the Co^II^ atom, as well as a non-coordinated caesium cation. The Co atom occupies a special position on a crystallographic inversion center. The caesium ion is octahedrally coordinated to six oxygen atoms with Cs—O distances ranging from 3.119 (2) to 3.296 (2) Å. The two methylene diphosphonate ligands chelate to the central cobalt metal *via* four oxygen atoms (O2/O2' and O7/O7') from the phosphonate groups. This leads to the formation of two six-membered rings.

The Co^II^ metal center has a slightly distorted octahedral geometry with O—Co—O angles ranging between 85.35 (8) ° and 94.65 (8) °. The Co—O bond lengths vary between 2.0761 (18) and 2.1272 (19) Å. These distances correspond to literature values (Bao *et al.* (2003); Cao *et al.* (2007); Gong *et al.* (2006); Van der Merwe *et al.* (2009); Visser *et al.* (2010); Yin *et al.* (2003).

A three-dimensional network is provided by O—H–O hydrogen bonds (Table 2).

### Experimental

[Co(NH_3_)_6_]Cl_3_(0,1700 g, 0,714 mmol) was dissolved in distilled water (10 cm^3^) and the pH of the solution was lowered to 1.87 using hydrochloric acid. The solution was heated for 30 minutes at 313.15 K. Methylene diphosphonate (0.251 g, 1.43 mmol) was dissolved in distilled water (7 cm^3^) and the pH of the solution was elevated to 1.93 using caesium chloride. Both solutions were combined and the pH was adjusted to 2.04, the pink solution was heated for 3 h at 353.15 K. Pink crystals, suitable for X-ray diffraction, was obtained. (Yield: 7.2%)

### Refinement

All H atoms were located from difference Fourier maps and were refined isotropically without further restraints. The highest residual electron density was located 0.88 Å from P1.

### Crystal data

**Table:**

Cs_2_[Co(CH_4_O_6_P_2_)_2_(H_2_O)_2_]	*Z* = 1
*M**_r_* = 708.75	*F*(000) = 253
Triclinic, *P*1	*D*_x_ = 2.953 Mg m^−^^3^
Hall symbol: -P 1	Mo *K*α radiation, λ = 0.71073 Å
*a* = 7.333 (5) Å	Cell parameters from 3160 reflections
*b* = 7.412 (5) Å	θ = 2.8–28.4°
*c* = 7.666 (5) Å	µ = 5.96 mm^−^^1^
α = 74.621 (5)°	*T* = 293 K
β = 83.064 (5)°	Needle, pink
γ = 86.496 (5)°	0.38 × 0.07 × 0.05 mm
*V* = 398.6 (5) Å^3^	

### Data collection

**Table:**

Bruker APEXII CCD diffractometer	1827 reflections with *I* > 2σ(*I*)
φ and ω scans	*R*_int_ = 0.015
Absorption correction: multi-scan (*SADABS*; Bruker, 2001)	θ_max_ = 28°, θ_min_ = 3.7°
*T*_min_ = 0.210, *T*_max_ = 0.755	*h* = −9→9
4203 measured reflections	*k* = −9→9
1904 independent reflections	*l* = −10→10

### Refinement

**Table:**

Refinement on *F*^2^	11 restraints
Least-squares matrix: full	H atoms treated by a mixture of independent and constrained refinement
*R*[*F*^2^ > 2σ(*F*^2^)] = 0.015	*w* = 1/[σ^2^(*F*_o_^2^) + (0.0412*P*)^2^ + 0.4899*P*] where *P* = (*F*_o_^2^ + 2*F*_c_^2^)/3
*wR*(*F*^2^) = 0.044	(Δ/σ)_max_ = 0.002
*S* = 0.75	Δρ_max_ = 0.47 e Å^−^^3^
1904 reflections	Δρ_min_ = −0.48 e Å^−^^3^
132 parameters	

### Special details

**Table:**

Geometry. All s.u.'s (except the s.u. in the dihedral angle between two l.s. planes) are estimated using the full covariance matrix. The cell s.u.'s are taken into account individually in the estimation of s.u.'s in distances, angles and torsion angles; correlations between s.u.'s in cell parameters are only used when they are defined by crystal symmetry. An approximate (isotropic) treatment of cell s.u.'s is used for estimating s.u.'s involving l.s. planes.

### Fractional atomic coordinates and isotropic or equivalent isotropic displacement parameters (Å^2^)

**Table:**

	*x*	*y*	*z*	*U*_iso_*/*U*_eq_	Occ. (<1)
Cs1	0.284577 (17)	0.954858 (18)	0.692901 (17)	0.00965 (6)	
Co1	0.5	0.5	0.5	0.00595 (9)	
P1	0.26392 (7)	0.34252 (8)	0.22866 (8)	0.00590 (11)	
P2	0.19616 (7)	0.74778 (8)	0.25147 (8)	0.00589 (11)	
O1	0.2886 (2)	0.4437 (2)	0.7201 (2)	0.0112 (3)	
O2	0.3857 (2)	0.3210 (2)	0.3786 (2)	0.0088 (3)	
O3	0.0667 (2)	0.2706 (2)	0.3122 (2)	0.0086 (3)	
O4	0.3336 (2)	0.2396 (2)	0.0861 (2)	0.0086 (3)	
O5	0.1822 (2)	0.9384 (2)	0.1036 (2)	0.0109 (3)	
O6	0.0107 (2)	0.7058 (2)	0.3621 (2)	0.0102 (3)	
O7	0.3541 (2)	0.7427 (2)	0.3623 (2)	0.0086 (3)	
C1	0.2397 (3)	0.5858 (3)	0.1121 (3)	0.0071 (4)	
H1A	0.313 (4)	0.383 (4)	0.837 (3)	0.023 (9)*	
H1B	0.185 (4)	0.410 (6)	0.704 (6)	0.063 (15)*	
H2	0.048 (5)	0.287 (5)	0.417 (3)	0.035 (10)*	
H3	0.145 (3)	0.599 (4)	0.033 (4)	0.018 (8)*	
H4	0.357 (3)	0.616 (4)	0.046 (4)	0.015 (8)*	
H5A	0.156 (9)	0.935 (9)	0.002 (5)	0.022*	0.5
H5B	0.239 (8)	1.030 (7)	0.116 (9)	0.022*	0.5

### Atomic displacement parameters (Å^2^)

**Table:**

	*U*^11^	*U*^22^	*U*^33^	*U*^12^	*U*^13^	*U*^23^
Cs1	0.01010 (9)	0.00910 (9)	0.00854 (9)	0.00056 (5)	−0.00040 (5)	−0.00068 (6)
Co1	0.00540 (19)	0.00593 (19)	0.0067 (2)	−0.00035 (15)	−0.00109 (15)	−0.00174 (16)
P1	0.0061 (2)	0.0053 (2)	0.0063 (3)	−0.00119 (19)	−0.0007 (2)	−0.0015 (2)
P2	0.0060 (2)	0.0060 (3)	0.0057 (3)	−0.00059 (19)	−0.0006 (2)	−0.0014 (2)
O1	0.0084 (8)	0.0160 (9)	0.0088 (8)	−0.0027 (7)	−0.0002 (6)	−0.0019 (7)
O2	0.0097 (7)	0.0077 (7)	0.0092 (8)	−0.0008 (6)	−0.0028 (6)	−0.0015 (6)
O3	0.0076 (7)	0.0110 (8)	0.0077 (8)	−0.0033 (6)	0.0007 (6)	−0.0032 (6)
O4	0.0098 (7)	0.0072 (7)	0.0096 (8)	−0.0012 (6)	0.0008 (6)	−0.0040 (6)
O5	0.0173 (8)	0.0063 (7)	0.0093 (8)	−0.0012 (6)	−0.0063 (7)	−0.0002 (6)
O6	0.0072 (7)	0.0139 (8)	0.0096 (8)	−0.0017 (6)	0.0012 (6)	−0.0040 (6)
O7	0.0096 (7)	0.0067 (7)	0.0098 (8)	−0.0003 (6)	−0.0032 (6)	−0.0016 (6)
C1	0.0087 (9)	0.0066 (10)	0.0056 (10)	−0.0009 (8)	−0.0011 (8)	−0.0005 (8)

### Geometric parameters (Å, °)

**Table:**

Cs1—O5^i^	3.119 (3)	P2—O6	1.5158 (18)
Cs1—O3^ii^	3.165 (2)	P2—O5	1.5663 (19)
Cs1—O2^iii^	3.162 (2)	P2—C1	1.799 (2)
Cs1—O2^iv^	3.173 (2)	O1—H1A	0.922 (17)
Cs1—O6^v^	3.192 (2)	O1—H1B	0.846 (19)
Cs1—O4^iii^	3.485 (2)	O2—Cs1^iii^	3.162 (2)
Cs1—O7^vi^	3.490 (2)	O2—Cs1^vii^	3.173 (2)
Co1—O2^iii^	2.0761 (18)	O3—Cs1^ii^	3.165 (2)
Co1—O2	2.0761 (18)	O3—H2	0.840 (19)
Co1—O1^iii^	2.1209 (19)	O4—Cs1^iii^	3.485 (2)
Co1—O1	2.1209 (19)	O5—Cs1^viii^	3.119 (3)
Co1—O7^iii^	2.1272 (19)	O5—H5A	0.83 (2)
Co1—O7	2.1272 (19)	O5—H5B	0.85 (2)
P1—O2	1.5087 (18)	O6—Cs1^v^	3.192 (2)
P1—O4	1.5158 (18)	O7—Cs1^vi^	3.490 (2)
P1—O3	1.5732 (18)	C1—H3	0.963 (14)
P1—C1	1.796 (3)	C1—H4	0.951 (14)
P2—O7	1.5099 (18)		
			
O5^i^—Cs1—O3^ii^	91.51 (5)	O7—Co1—Cs1^iii^	128.78 (6)
O5^i^—Cs1—O2^iii^	113.53 (4)	O2^iii^—Co1—Cs1^vi^	45.80 (6)
O3^ii^—Cs1—O2^iii^	103.25 (7)	O2—Co1—Cs1^vi^	134.20 (6)
O5^i^—Cs1—O2^iv^	125.59 (5)	O1^iii^—Co1—Cs1^vi^	60.18 (5)
O3^ii^—Cs1—O2^iv^	120.31 (5)	O1—Co1—Cs1^vi^	119.82 (5)
O2^iii^—Cs1—O2^iv^	101.31 (5)	O7^iii^—Co1—Cs1^vi^	125.13 (6)
O5^i^—Cs1—O6^v^	82.95 (5)	O7—Co1—Cs1^vi^	54.87 (6)
O3^ii^—Cs1—O6^v^	81.36 (7)	Cs1^iii^—Co1—Cs1^vi^	123.10 (4)
O2^iii^—Cs1—O6^v^	162.47 (4)	O2^iii^—Co1—Cs1^vii^	134.20 (6)
O2^iv^—Cs1—O6^v^	62.52 (5)	O2—Co1—Cs1^vii^	45.80 (6)
O5^i^—Cs1—O4^iii^	73.40 (5)	O1^iii^—Co1—Cs1^vii^	119.82 (5)
O3^ii^—Cs1—O4^iii^	124.24 (6)	O1—Co1—Cs1^vii^	60.18 (5)
O2^iii^—Cs1—O4^iii^	44.75 (5)	O7^iii^—Co1—Cs1^vii^	54.87 (6)
O2^iv^—Cs1—O4^iii^	111.44 (5)	O7—Co1—Cs1^vii^	125.13 (6)
O6^v^—Cs1—O4^iii^	144.68 (4)	Cs1^iii^—Co1—Cs1^vii^	56.90 (4)
O5^i^—Cs1—O7^vi^	94.14 (4)	Cs1^vi^—Co1—Cs1^vii^	180
O3^ii^—Cs1—O7^vi^	170.61 (4)	O2—P1—O4	114.76 (10)
O2^iii^—Cs1—O7^vi^	81.34 (7)	O2—P1—O3	109.86 (10)
O2^iv^—Cs1—O7^vi^	50.37 (5)	O4—P1—O3	107.73 (9)
O6^v^—Cs1—O7^vi^	91.89 (7)	O2—P1—C1	109.55 (10)
O4^iii^—Cs1—O7^vi^	64.74 (6)	O4—P1—C1	107.05 (11)
O5^i^—Cs1—C1^i^	44.75 (6)	O3—P1—C1	107.62 (10)
O3^ii^—Cs1—C1^i^	72.92 (5)	O2—P1—Cs1^iii^	51.16 (7)
O2^iii^—Cs1—C1^i^	78.47 (6)	O4—P1—Cs1^iii^	63.66 (7)
O2^iv^—Cs1—C1^i^	165.97 (5)	O3—P1—Cs1^iii^	124.40 (8)
O6^v^—Cs1—C1^i^	118.91 (5)	C1—P1—Cs1^iii^	127.77 (8)
O4^iii^—Cs1—C1^i^	58.52 (5)	O2—P1—Cs1^vii^	49.06 (7)
O7^vi^—Cs1—C1^i^	116.25 (5)	O4—P1—Cs1^vii^	103.57 (8)
O5^i^—Cs1—O1^iv^	74.68 (4)	O3—P1—Cs1^vii^	68.88 (7)
O3^ii^—Cs1—O1^iv^	126.32 (5)	C1—P1—Cs1^vii^	148.54 (8)
O2^iii^—Cs1—O1^iv^	130.04 (5)	Cs1^iii^—P1—Cs1^vii^	61.70 (2)
O2^iv^—Cs1—O1^iv^	50.97 (5)	O7—P2—O6	114.83 (11)
O6^v^—Cs1—O1^iv^	46.06 (5)	O7—P2—O5	111.69 (10)
O4^iii^—Cs1—O1^iv^	101.41 (4)	O6—P2—O5	109.44 (10)
O7^vi^—Cs1—O1^iv^	48.72 (4)	O7—P2—C1	110.57 (10)
C1^i^—Cs1—O1^iv^	118.60 (6)	O6—P2—C1	108.09 (10)
O5^i^—Cs1—O3^iv^	125.42 (5)	O5—P2—C1	101.32 (11)
O3^ii^—Cs1—O3^iv^	78.65 (6)	O7—P2—Cs1^v^	124.05 (8)
O2^iii^—Cs1—O3^iv^	121.02 (5)	O5—P2—Cs1^v^	65.52 (7)
O2^iv^—Cs1—O3^iv^	42.33 (5)	C1—P2—Cs1^v^	125.05 (8)
O6^v^—Cs1—O3^iv^	42.63 (4)	O7—P2—Cs1^viii^	118.54 (7)
O4^iii^—Cs1—O3^iv^	152.49 (4)	O6—P2—Cs1^viii^	125.77 (7)
O7^vi^—Cs1—O3^iv^	91.97 (5)	C1—P2—Cs1^viii^	61.03 (8)
C1^i^—Cs1—O3^iv^	148.87 (5)	Cs1^v^—P2—Cs1^viii^	94.916 (19)
O1^iv^—Cs1—O3^iv^	69.55 (5)	Co1—O1—Cs1^vii^	89.88 (6)
O5^i^—Cs1—O5^v^	55.64 (5)	Co1—O1—H1A	122 (2)
O3^ii^—Cs1—O5^v^	56.50 (6)	Cs1^vii^—O1—H1A	81 (2)
O2^iii^—Cs1—O5^v^	153.15 (4)	Co1—O1—H1B	120 (3)
O2^iv^—Cs1—O5^v^	104.38 (5)	Cs1^vii^—O1—H1B	66 (3)
O6^v^—Cs1—O5^v^	41.87 (4)	H1A—O1—H1B	108 (3)
O4^iii^—Cs1—O5^v^	128.47 (5)	P1—O2—Co1	135.98 (10)
O7^vi^—Cs1—O5^v^	121.54 (5)	P1—O2—Cs1^iii^	107.03 (9)
C1^i^—Cs1—O5^v^	78.50 (5)	Co1—O2—Cs1^iii^	103.97 (7)
O1^iv^—Cs1—O5^v^	73.92 (4)	P1—O2—Cs1^vii^	109.88 (8)
O3^iv^—Cs1—O5^v^	75.37 (5)	Co1—O2—Cs1^vii^	106.22 (8)
O5^i^—Cs1—P1^iii^	94.28 (4)	Cs1^iii^—O2—Cs1^vii^	78.69 (5)
O3^ii^—Cs1—P1^iii^	115.59 (6)	P1—O3—Cs1^ii^	150.94 (9)
O2^iii^—Cs1—P1^iii^	21.82 (3)	P1—O3—Cs1^vii^	87.66 (8)
O2^iv^—Cs1—P1^iii^	106.97 (4)	Cs1^ii^—O3—Cs1^vii^	101.35 (6)
O6^v^—Cs1—P1^iii^	162.96 (3)	P1—O3—H2	108 (3)
O4^iii^—Cs1—P1^iii^	22.94 (3)	Cs1^ii^—O3—H2	101 (3)
O7^vi^—Cs1—P1^iii^	71.49 (6)	Cs1^vii^—O3—H2	59 (3)
C1^i^—Cs1—P1^iii^	67.80 (4)	P1—O4—Cs1^iii^	93.39 (8)
O1^iv^—Cs1—P1^iii^	116.98 (4)	P2—O5—Cs1^viii^	120.07 (9)
O3^iv^—Cs1—P1^iii^	138.50 (3)	P2—O5—Cs1^v^	91.93 (8)
O5^v^—Cs1—P1^iii^	145.78 (4)	Cs1^viii^—O5—Cs1^v^	124.36 (5)
O2^iii^—Co1—O2	180	P2—O5—H5A	118 (5)
O2^iii^—Co1—O1^iii^	90.76 (8)	Cs1^v^—O5—H5A	99 (5)
O2—Co1—O1^iii^	89.24 (8)	P2—O5—H5B	117 (5)
O2^iii^—Co1—O1	89.24 (8)	Cs1^viii^—O5—H5B	103 (5)
O2—Co1—O1	90.76 (8)	Cs1^v^—O5—H5B	100 (5)
O1^iii^—Co1—O1	180	H5A—O5—H5B	121 (6)
O2^iii^—Co1—O7^iii^	94.65 (8)	P2—O6—Cs1^v^	115.52 (9)
O2—Co1—O7^iii^	85.35 (8)	P2—O7—Co1	126.62 (10)
O1^iii^—Co1—O7^iii^	91.47 (7)	P2—O7—Cs1^vi^	131.62 (9)
O1—Co1—O7^iii^	88.53 (7)	Co1—O7—Cs1^vi^	95.23 (7)
O2^iii^—Co1—O7	85.35 (8)	P1—C1—P2	116.79 (13)
O2—Co1—O7	94.65 (8)	P1—C1—Cs1^viii^	149.47 (11)
O1^iii^—Co1—O7	88.53 (7)	P2—C1—Cs1^viii^	93.21 (10)
O1—Co1—O7	91.47 (7)	P1—C1—H3	108.1 (19)
O7^iii^—Co1—O7	180.00 (9)	P2—C1—H3	110.1 (19)
O2^iii^—Co1—Cs1^iii^	132.78 (6)	Cs1^viii^—C1—H3	62.8 (19)
O2—Co1—Cs1^iii^	47.22 (6)	P1—C1—H4	104.3 (19)
O1^iii^—Co1—Cs1^iii^	63.15 (5)	P2—C1—H4	105.3 (19)
O1—Co1—Cs1^iii^	116.85 (5)	Cs1^viii^—C1—H4	59.3 (18)
O7^iii^—Co1—Cs1^iii^	51.22 (6)	H3—C1—H4	112 (3)
			
O2^iii^—Co1—O1—Cs1^vii^	−144.21 (6)	C1—P2—O5—Cs1^viii^	−8.70 (12)
O2—Co1—O1—Cs1^vii^	35.79 (6)	Cs1^v^—P2—O5—Cs1^viii^	−132.22 (9)
O7^iii^—Co1—O1—Cs1^vii^	−49.55 (6)	O7—P2—O5—Cs1^v^	−118.75 (9)
O7—Co1—O1—Cs1^vii^	130.45 (6)	O6—P2—O5—Cs1^v^	9.54 (9)
Cs1^iii^—Co1—O1—Cs1^vii^	−5.22 (5)	C1—P2—O5—Cs1^v^	123.53 (8)
Cs1^vi^—Co1—O1—Cs1^vii^	180	Cs1^viii^—P2—O5—Cs1^v^	132.22 (9)
O4—P1—O2—Co1	130.04 (13)	O7—P2—O6—Cs1^v^	114.18 (10)
O3—P1—O2—Co1	−108.42 (14)	O5—P2—O6—Cs1^v^	−12.34 (11)
C1—P1—O2—Co1	9.60 (17)	C1—P2—O6—Cs1^v^	−121.88 (10)
Cs1^iii^—P1—O2—Co1	132.87 (17)	Cs1^viii^—P2—O6—Cs1^v^	−55.00 (10)
Cs1^vii^—P1—O2—Co1	−143.29 (18)	O6—P2—O7—Co1	80.65 (14)
O4—P1—O2—Cs1^iii^	−2.83 (11)	O5—P2—O7—Co1	−154.00 (11)
O3—P1—O2—Cs1^iii^	118.71 (9)	C1—P2—O7—Co1	−41.97 (15)
C1—P1—O2—Cs1^iii^	−123.27 (9)	Cs1^v^—P2—O7—Co1	131.64 (9)
Cs1^vii^—P1—O2—Cs1^iii^	83.84 (8)	Cs1^viii^—P2—O7—Co1	−109.34 (10)
O4—P1—O2—Cs1^vii^	−86.67 (11)	O6—P2—O7—Cs1^vi^	−135.06 (11)
O3—P1—O2—Cs1^vii^	34.87 (11)	O5—P2—O7—Cs1^vi^	−9.70 (14)
C1—P1—O2—Cs1^vii^	152.89 (9)	C1—P2—O7—Cs1^vi^	102.32 (13)
Cs1^iii^—P1—O2—Cs1^vii^	−83.84 (8)	Cs1^v^—P2—O7—Cs1^vi^	−84.06 (12)
O1^iii^—Co1—O2—P1	−80.87 (15)	Cs1^viii^—P2—O7—Cs1^vi^	34.96 (13)
O1—Co1—O2—P1	99.13 (15)	O2^iii^—Co1—O7—P2	−168.93 (12)
O7^iii^—Co1—O2—P1	−172.41 (15)	O2—Co1—O7—P2	11.07 (12)
O7—Co1—O2—P1	7.59 (15)	O1^iii^—Co1—O7—P2	100.19 (13)
Cs1^iii^—Co1—O2—P1	−133.77 (17)	O1—Co1—O7—P2	−79.81 (13)
Cs1^vi^—Co1—O2—P1	−35.83 (17)	Cs1^iii^—Co1—O7—P2	47.08 (14)
Cs1^vii^—Co1—O2—P1	144.17 (17)	Cs1^vi^—Co1—O7—P2	154.02 (14)
O1^iii^—Co1—O2—Cs1^iii^	52.90 (7)	Cs1^vii^—Co1—O7—P2	−25.98 (14)
O1—Co1—O2—Cs1^iii^	−127.10 (7)	O2^iii^—Co1—O7—Cs1^vi^	37.05 (6)
O7^iii^—Co1—O2—Cs1^iii^	−38.64 (6)	O2—Co1—O7—Cs1^vi^	−142.95 (6)
O7—Co1—O2—Cs1^iii^	141.36 (6)	O1^iii^—Co1—O7—Cs1^vi^	−53.82 (6)
Cs1^vi^—Co1—O2—Cs1^iii^	97.94 (6)	O1—Co1—O7—Cs1^vi^	126.18 (6)
Cs1^vii^—Co1—O2—Cs1^iii^	−82.06 (6)	Cs1^iii^—Co1—O7—Cs1^vi^	−106.94 (4)
O1^iii^—Co1—O2—Cs1^vii^	134.96 (7)	Cs1^vii^—Co1—O7—Cs1^vi^	180
O1—Co1—O2—Cs1^vii^	−45.04 (7)	O2—P1—C1—P2	−44.78 (16)
O7^iii^—Co1—O2—Cs1^vii^	43.42 (6)	O4—P1—C1—P2	−169.80 (12)
O7—Co1—O2—Cs1^vii^	−136.58 (6)	O3—P1—C1—P2	74.63 (14)
Cs1^iii^—Co1—O2—Cs1^vii^	82.06 (6)	Cs1^iii^—P1—C1—P2	−100.25 (12)
Cs1^vi^—Co1—O2—Cs1^vii^	180	Cs1^vii^—P1—C1—P2	−3.5 (2)
O2—P1—O3—Cs1^ii^	−136.98 (17)	O2—P1—C1—Cs1^viii^	123.58 (19)
O4—P1—O3—Cs1^ii^	−11.3 (2)	O4—P1—C1—Cs1^viii^	−1.4 (2)
C1—P1—O3—Cs1^ii^	103.8 (2)	O3—P1—C1—Cs1^viii^	−117.01 (19)
Cs1^iii^—P1—O3—Cs1^ii^	−81.1 (2)	Cs1^iii^—P1—C1—Cs1^viii^	68.1 (2)
Cs1^vii^—P1—O3—Cs1^ii^	−109.40 (19)	Cs1^vii^—P1—C1—Cs1^viii^	164.84 (9)
O2—P1—O3—Cs1^vii^	−27.58 (9)	O7—P2—C1—P1	62.05 (15)
O4—P1—O3—Cs1^vii^	98.08 (9)	O6—P2—C1—P1	−64.42 (15)
C1—P1—O3—Cs1^vii^	−146.80 (8)	O5—P2—C1—P1	−179.41 (12)
Cs1^iii^—P1—O3—Cs1^vii^	28.30 (7)	Cs1^v^—P2—C1—P1	−111.48 (11)
O2—P1—O4—Cs1^iii^	2.46 (9)	Cs1^viii^—P2—C1—P1	174.11 (15)
O3—P1—O4—Cs1^iii^	−120.24 (8)	O7—P2—C1—Cs1^viii^	−112.06 (8)
C1—P1—O4—Cs1^iii^	124.27 (8)	O6—P2—C1—Cs1^viii^	121.47 (8)
Cs1^vii^—P1—O4—Cs1^iii^	−48.42 (5)	O5—P2—C1—Cs1^viii^	6.48 (9)
O7—P2—O5—Cs1^viii^	109.03 (10)	Cs1^v^—P2—C1—Cs1^viii^	74.41 (7)
O6—P2—O5—Cs1^viii^	−122.68 (10)		

Symmetry codes: (i) *x*, *y*, *z*+1; (ii) −*x*, −*y*+1, −*z*+1; (iii) −*x*+1, −*y*+1, −*z*+1; (iv) *x*, *y*+1, *z*; (v) −*x*, −*y*+2, −*z*+1; (vi) −*x*+1, −*y*+2, −*z*+1; (vii) *x*, *y*−1, *z*; (viii) *x*, *y*, *z*−1.

### Hydrogen-bond geometry (Å, °)

**Table:**

*D*—H···*A*	*D*—H	H···*A*	*D*···*A*	*D*—H···*A*
O1—H1A···O4^i^	0.92 (2)	1.94 (2)	2.860 (3)	173 (3)
O5—H5B···O4^iv^	0.85 (2)	1.69 (3)	2.518 (3)	164 (7)

Symmetry codes: (i) *x*, *y*, *z*+1; (iv) *x*, *y*+1, *z*.
